# Molecular Changes Underlying Hypertrophic Scarring Following Burns Involve Specific Deregulations at All Wound Healing Stages (Inflammation, Proliferation and Maturation)

**DOI:** 10.3390/ijms22020897

**Published:** 2021-01-18

**Authors:** Matúš Čoma, Lucia Fröhlichová, Lukáš Urban, Robert Zajíček, Tomáš Urban, Pavol Szabo, Štěpán Novák, Vitaly Fetissov, Barbora Dvořánková, Karel Smetana, Peter Gál

**Affiliations:** 1 Department of Biomedical Research, East-Slovak Institute of Cardiovascular Diseases, Inc., 040 11 Košice, Slovakia; mcoma@vusch.sk (M.Č.); lurban@vusch.sk (L.U.); 2 Department of Pharmacology, Faculty of Medicine, Pavol Jozef Šafárik University, 040 11 Košice, Slovakia; 3 Department of Pathology, Louise Pasteur University Hospital, 041 90 Košice, Slovakia; lucia.frohlichova@unlp.sk; 4 Center of Clinical and Preclinical Research MediPark, Pavol Jozef Šafárik University, 040 11 Košice, Slovakia; 5 Prague Burn Centre, Third Faculty of Medicine, Charles University and University Hospital Královské Vinohrady, 100 34 Prague, Czech Republic; robert.zajicek@lf3.cuni.cz (R.Z.); tomas.urban@lf3.cuni.cz (T.U.); vitaly.fetissov@fnkv.cz (V.F.); 6 Institute of Anatomy, First Faculty of Medicine, Charles University, 128 00 Prague, Czech Republic; pavol.szabo@lf1.cuni.cz (P.S.); stepan.novak@lf1.cuni.cz (Š.N.); barbora.dvorankova@lf1.cuni.cz (B.D.); 7 BIOCEV, 252 50 Vestec, Czech Republic; 8 Department of Otorhinolaryngology, Head and Neck Surgery, First Faculty of Medicine, Charles University and University Hospital Motol, 150 06 Prague, Czech Republic

**Keywords:** wound healing, skin, burn, pathological scar, cell interaction, stem cell

## Abstract

Excessive connective tissue accumulation, a hallmark of hypertrophic scaring, results in progressive deterioration of the structure and function of organs. It can also be seen during tumor growth and other fibroproliferative disorders. These processes result from a wide spectrum of cross-talks between mesenchymal, epithelial and inflammatory/immune cells that have not yet been fully understood. In the present review, we aimed to describe the molecular features of fibroblasts and their interactions with immune and epithelial cells and extracellular matrix. We also compared different types of fibroblasts and their roles in skin repair and regeneration following burn injury. In summary, here we briefly review molecular changes underlying hypertrophic scarring following burns throughout all basic wound healing stages, i.e. during inflammation, proliferation and maturation.

## 1. Introduction

**Figure 1:**
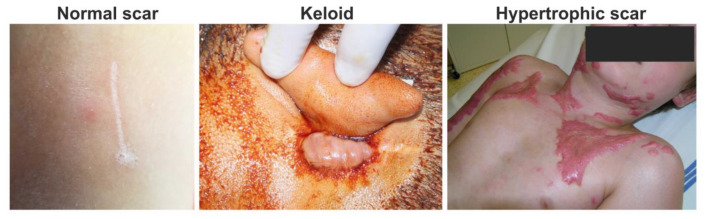
Clinical photos of normal, keloid and hypertrophic scars: normal fine line scar following a surgical wound (**left**); keloid scar, which extends beyond the original wound (**middle**); and hypertrophic scar, which does not extend beyond the initial site of the skin lesion associated with burn injury (**right**).

Each year, over 100 million patients in the developed world form scars as a result of surgery and/or trauma [1]. Hypotrophic, atrophic (e.g., acne scars) and linear normotrophic maturated scars have little to no effect on the patient’s quality of life (Figure 1) [2]. However, pruritus, pain and contractures often related to extreme scarring, such as hypertrophic or keloid scarring, dramatically affect the patient’s physical and psychological state [3,4,5]. In particular, hypertrophic and keloid scarring are of the highest clinical relevance in burn treatment and esthetic medicine [1]. Since hypertrophic and keloid scarring belong to the family of fibroproliferative disorders, these scars greatly differ from hypotrophic, atrophic and normotrophic scars in their histological features [6]. Clinical differentiation between hypertrophic and keloid scars is often challenging. Proper identification of the scar type is necessary for appropriate management of scar formation and cosmetic surgery approach [7]. Insight into the complex molecular mechanisms involved in extreme scarring reviewed in this article is the key to understand the complicated pathophysiology of excessive scarring and related fibroproliferative disorders. In this review, we focus on fibroblasts, the key cellular component in wound healing. We compare their function in normal and hypertrophic wound healing during chronic inflammation, as well as their production and remodeling of extracellular matrix (ECM).

## 2. Inflammatory Phase of Wound Healing

**Figure 2:**
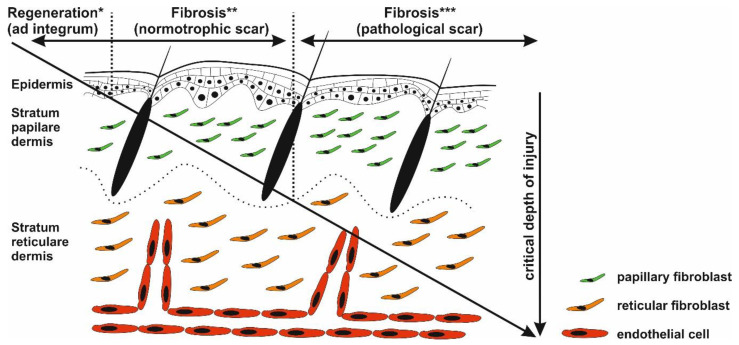
Critical depth of injury. Only the epidermis has the capability to fully regenerate (*), whereas healing of the injured dermis results in scar formation (** and ***). Injury targeting only papillary dermis ends with normotrophic scar formation (*). Deeper wounds (reticular parts of the dermis) with prolonged inflammatory response may activate biological pathways resulting in pathological scarring (***).

Wound healing is a very complex process involving activation of different cell types, production of various cytokines, chemokines and many factors affecting immune responses, and is thus prone to errors [8]. Possibly arising imbalances in proinflammatory and anti-inflammatory signaling molecules lead to an excessively strong and prolonged inflammatory response causing pathological scarring [9,10]. Such scarring was observed following cutaneous injury or/and irritation, including trauma, insect bite, burn injury grade 2b, surgery, vaccination, skin piercing, acne, and virus infections. On the other hand, pathological scarring was not observed following superficial injuries not reaching reticular dermis (Figure 2), suggesting a critical role of deeply localized inflammation [11]. Even though inflammatory responses begin immediately after injury, keloid and hypertrophic scars may form weeks or even years after injury [12]. By this time, the wound is closed and the epidermis fully regenerated. The pathological scar therefore results as a product of chronic inflammation of reticular dermis and continuous aberrant dermal matrix production [11,13]. During the chronic phase, inflammation of reticular dermis and subsequent scarring can be further accelerated by recurrent trauma or mechanical damage to the dermis layer [14,15,16]. In general, long and systemic inflammation persistency is typical of burn injuries, particularly in injured patients with severe skin loss and/or deep skin defects [17].

It has been generally accepted that skin wound healing runs in four characteristic stages: hemostasis, inflammation, proliferation, and maturation/remodeling. The initial phase called hemostasis, preventing blood loss, is immediately followed by the inflammatory phase, and therefore sometimes referred to as vascular reaction of the inflammatory phase [18]. Initial blood leakage triggers activation of otherwise inert zymogenes resulting in platelet activation and fibrin clot formation [19]. At the site of injury, platelets are stimulated by contact with subendothelium, tissue factors and collagens, leading to their degranulation [20] and release of proinflammatory molecules typical of initial wound healing phase involving interleukins (e.g., IL-4, IL-5, IL-6, IL-8, IL-13), chemokines (e.g., CXCL-4, CXCL8), growth factors (e.g., TGF-β1, TGF-β2, PDGF, IGF, EGF, VEGF, FGF), and TIMP-1 and TIMP-2 [21,22,23,24,25]. On the other hand, expression of antifibrotic factors is diminished (e.g., IFN-γ, IL-10, IL-12, TNF-α). Importantly, the formed fibrin clot represents a permeable structure, which further facilitates migration of cells involved in wound healing, namely keratinocytes, immune cells and fibroblasts [26,27]. Of note, fibroblasts isolated from keloids express higher levels of chemokine receptors, making them more sensitive to migration stimuli [6].

### Immune Cells

**Figure 3:**
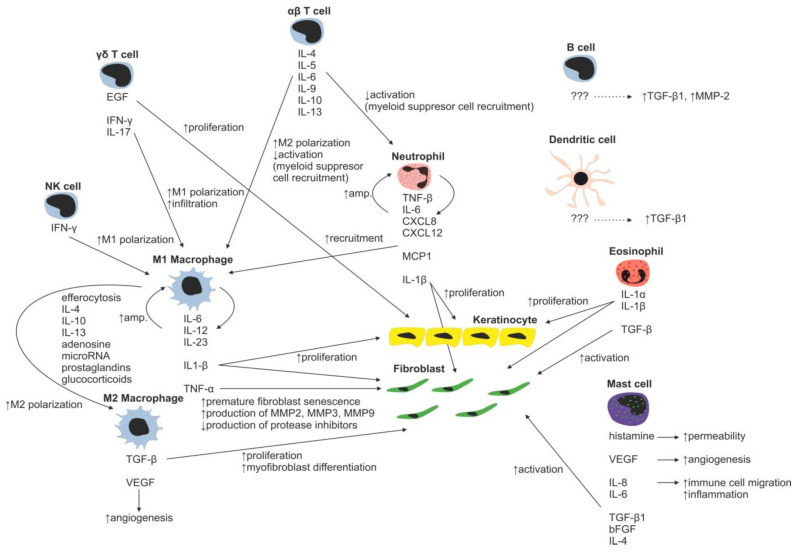
Immune cell regulation of normal wound healing.

**Figure 4:**
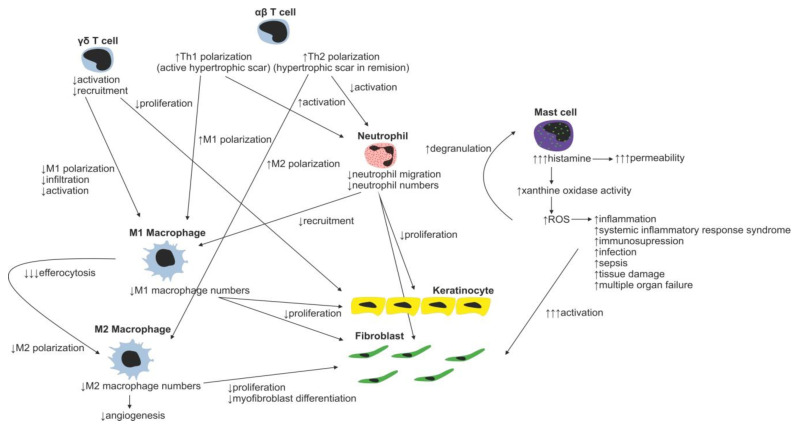
Changes in immune cell regulation of wound healing following burn injury.

Neutrophils with over 30 different receptors responding to chemokines and other activation molecules (e.g., IL-8 and CXCL-8) are the first wound-infiltrating immune cells [28,29]. They represent the most abundant immune cell type responsible for pathogen and debris removal (forming the demarcation line separating dead/infected tissue from vital/healthy tissue) [30]. Neutrophils are capable of immune response amplification by recruitment of additional neutrophils (Figure 3) (by releasing TNF-α, IL-1β, IL-6, CXCL8, and CXL12) and macrophages (by releasing monocyte chemoattractant protein 1—MCP-1). Proteases secreted by neutrophils are able to degrade TGF-β1 and PDGF, thereby arresting wound proliferation and remodeling processes [31]. In normal wounds, abundant neutrophils, which have completed their tasks, undergo apoptosis and are removed by local macrophages, resulting into inflammatory response attenuation [32,33]. However, in burn wounds, neutrophils hardly reach the site of injury due to the damage of blood vessels and lack of blood flow (Figure 4). Reduction in the average neutrophil migration speed [34] and low oxygen pressure [35] further complicates neutrophil navigation towards pathogens, resulting in a prolonged healing process in burned patients.

During normal skin wound healing, neutrophils are replaced by macrophages within a few days. The major source of wound-related macrophages is the pool of circulating monocytes in the blood vessels, which enter the injury site through vascular leakage, whereas skin-residing macrophages represent the minor source [36,37,38]. At the injury site, residing macrophages are polarized to inflammatory phenotype, sometimes referred to as M1 phenotype, as a response to the local presence of pathogens, necrotic tissue residues and various factors such as natural killer cell derived IFN-γ [39,40]. M1 macrophages secrete proinflammatory cytokines IL-1β, IL-6, IL-12, IL-23, and TNF-α, which further stimulate infiltrating monocytes [41,42,43,44]). The secreted IL-1 increases collagen synthesis and promotes fibroblast and keratinocyte proliferation [45]. However, M1-secreted TNF-α is linked to premature fibroblast senescence. Senescent fibroblasts contribute to wound chronicity by overproduction of proteases (e.g., MMP-2, MMP-3 and MMP-9) and decreased production of protease inhibitors [46]. To terminate inflammation during normal wound healing, a crucial transition of macrophages from proinflammatory M1 to anti-inflammatory M2 phenotype has to occur. A major contributor to the M2 phenotype polarization is efferocytosis, the process of used and abundant neutrophil phagocytosis by macrophages, which is further facilitated by other mediators such as prostaglandins, glucocorticoids, IL-4, IL-10, IL-13, adenosine, and microRNAs [47,48,49,50,51]. M2 macrophages have strong inflammation-resolving effects mediated by secretion of anti-inflammatory cytokines (e.g., IL-1 receptor antagonist, IL-10) and fibroblast proliferation and angiogenesis-stimulating effects by production of TGF-β and VEGF [52].

It is known that lack of macrophages, characteristically present in burns, negatively affects wound healing [53]. Not only are macrophages unable to reach the injury site analogically to neutrophils, the lack of neutrophils leads to imbalance in spent neutrophil efferocytosis, which in turn negatively affects macrophage M2 polarization and results in prolonged and/or unresolved chronic inflammation, delaying initiation of the proliferation phase [54]. Of note, macrophages may be polarized towards the M1 phenotype prior to entering the injury site as a response to altered wound microenvironment (WME). This prepolarization was also observed in nonhealing diabetic wounds (hyperglycemic conditions) and/or in patients with arterial/venous insufficiency (hypoxic conditions) with persisting chronic venous disease [54,55,56].

The role of various T-cell subsets throughout the wound-healing process and scar formation is much more intriguing. Epidermal γδ T cells residing at the injury site react to the tissue damage immediately by releasing epithelial growth factors and proinflammatory cytokines (IFN-γ, IL-17) [57], stimulating keratinocyte proliferation and immune cell infiltration. In normal wounds, γδ T cells contribute to early wound closure, whereas an absence of γδ T cells leads to impaired healing [58]. On the other hand, γδ T cell derived proinflammatory (IFN-γ, IL-17) [59,60] cytokines promote early macrophage infiltration, which is associated with greater scar size, and thus it was hypothesized that γδ T cells indirectly increase scarring [61]. In burns, γδ T cell activation and recruitment are suppressed, leading to insufficient macrophage activation resulting in poor healing of the burn wound. Thus, the γδ T cell reactivation therapeutic approach has already been proposed in burn patients [62].

Contrary to γδ T cells, αβ T cells, another T-cell subset, play an important role in transition from the inflammatory to tissue maturation/remodeling phase of wound healing. αβ T cells produce Th2 cytokines (e.g., IL-4, IL-5, IL-6, IL-9, IL-10 and IL-13) attenuating early inflammatory response by suppression of myeloid cell activation and myeloid suppressor cell recruitment [63], thus promoting Th2 polarization of T-cell subsets in WME. In normally healing wounds, the Th1 polarized T cells were shown to be predominantly present [64]. By contrast, in burn patients, the Th1 polarized T cells were found in active hypertrophic scars, whereas the Th2 phenotype was characteristic for the remission phase of hypertrophic scar formation [65]. In general, as a result of the postburn systemic response, an increase in serum levels of inflammatory cytokines TNF-α, IL-8 and IL-6 is present in burned patients [66]. Moreover, it was shown that serum levels of IL-6, an interleukin strongly promoting Th2 differentiation [67], positively correlate with the severity of burn injury [68]. Even though the mechanism promoting Th2 polarization of T cells is rather multifactorial, the aforementioned αβ T cells together with systemic response are probably the strongest contributors promoting this process. The impact of the systemic response on the T-cell population is remarkably evident three to seven days following a burn injury as a significant reduction in both CD4+ and CD8+ T-cell populations [69]. Furthermore, a decreased CD4:CD8 T-cell ratio was observed in burn wounds, most probably as a result of αβ T-cell involvement in the burn wound pathogenesis [63]. A decrease in total T-cell numbers, diminished CD4:CD8 T-cell ratio, as well as anti-inflammatory Th2 T-cell polarization result in reduction of the T-cell dependent immune function in burn wounds [70]. Thus, the αβ T-cell population has been proposed as an important therapeutic target for improving wound healing and recovery in burn patients [63].

Another well-studied immune cell population involved in the process of wound healing includes mast cells. Post-traumatically activated mast cells produce histamine, VEGF, IL-6 and IL-8, and thus increase endothelial permeability and facilitate migration of immune cells. Releasing IL-4, TGF-β1 and bFGF, they activate fibroblasts to produce and organize collagen fibrils [71,72]. Moreover, mast cells are able to stimulate fibroblasts to myofibroblast differentiation, gap junction formation and ECM contraction [73,74,75]. Inhibition of mast cell activation in a murine wound-healing model resulted in reduced scar width and higher fibrillar density of ECM [76]. For example, second trimester fetal wounds, wounds that heal without scarring, contain less dermal mast cells with the absence of degranulation compared to third semester wounds [77]. Therefore, the high number of mast cells observed in hypertrophic scars, compared to normal scars, may present a potential therapeutic target to face the abundant scar tissue production [72]. Burn injury stimulates mast cells to produce enormous amounts of histamine, which in turn enhances xanthine oxidase production of reactive oxygen species (ROS). ROS present in burns further strongly stimulate mast cell degranulation [78]. Huge production of ROS is dangerous due to its implications in inflammation, systemic inflammatory response syndrome, immunosuppression, infection and sepsis, tissue damage and multiple organ failure [79].

So far, only a few papers studying the role of dendritic and B cells in burn scar formation have appeared. B cells, when topically applied, increase TGF-β and decrease MMP2 expressions in granulation tissue, improve healing, reduce scar size, increase collagen deposition and maturation, enhance angiogenesis, increase fibroblast proliferation and alter neutrophil infiltration [80,81]. Interestingly, it was shown that B-cell levels were significantly increased up to three and seven days post burn injury compared with healthy controls [69]. In this line of evidence, enhanced inflation of dendritic cells led to significantly accelerated wound closure, enhanced angiogenesis and cellular proliferation associated with increased TGF-β1 levels. Similarly to B cells, wound enhancement with dendritic cells was suggested as a promising new therapeutic approach in burn injury treatment [80,82].

So far, the role of other immune cell types in burn wound healing has not been comprehensively studied. For example, eosinophils secrete interleukins/growth factors IL-1α, IL-1β, TGF-β1 promoting keratinocyte migration and are able to stimulate fibroblasts to upregulate α-smooth muscle actin expression and collagen secretion [83]. However, the extent and molecular mechanism of the resulting effect of these cells on burn scar formation needs to be elucidated in further studies.

## 3. Proliferation Phase of Wound Healing

After pathogen removal and wound cleaning, the initial hemostasis/inflammation phase is followed by the proliferation phase, which usually occurs from days 4 to 21 [84] and is characterized by formation of granulation tissue. Initially, the recovering tissue is full of proliferating fibroblasts producing loose fibronectin-rich extracellular matrix [85]. Firstly formed collagen type III is slowly replaced during the maturation phase by collagen type I [86]. Finally, differentiated fibroblasts, myofibroblasts, accelerate wound closure by pulling wound margins together, mimicking action of smooth muscle cells [87]. In the meantime, epithelial cells migrate from the wound base to its top reproducing epidermis, a critical protective barrier, in a process also known as re-epithelialization [88]. The three key cell populations involved in the above-mentioned processes are fibroblasts/endothelial cells and keratinocytes, whose mutual interactions also known as epithelial-mesenchymal cross-talk are crucial for WME formation and wound healing outcome. Impairments in their interactions lead to excessive fibrotic scar formation and/or poor wound healing.

### 3.1. Epidermis Regeneration

Epidermis, the upper layer of the skin, is formed by multilayer growth of keratinocytes, which is continuously renewing itself. Keratinocytes originate from stem cells and mainly progenitor (transit-amplifying) cells located in the basal layer of the epidermis. These cells are characterized by expression of keratins 5 and 14 [89]. Stem cells, besides the basal layer, are also localized in the sweat glands and mainly in pilosebaceous units, in the region called bulge [90]. Epidermal stem cells are slow-cycling, label-retaining cells characterized by high expression of β1-integrin, keratin 19, and absence of CD71 [91]. In partial-thickness wounds, where the deep dermal structures are preserved, stem cells from the bulge and sweat glands are responsible for the resurfacing of wounds resulting in fast and even re-epithelization [92]. In full-thickness wounds, where the whole dermis is completely destroyed, re-epithelization is restricted to migrating keratinocytes from the wound margins. The entire process of re-epithelization includes several basic steps. At the beginning, formation of provisional extracellular matrix by insoluble proteins is followed by migration, proliferation and stratification of keratinocytes. Already 24 h after injury, the activation of keratinocytes leads to hyperproliferative migratory phenotype characterized by expression of keratins 6, 16 and 17 [93]. Paracrine release of IL-1 triggers activation of keratinocytes, which are maintained in activated state by production of TNF-α [90]. Complete epidermis regeneration is characterized by reconstruction of dermo-epidermal junctions creating a barrier between the inner and outer environment [94], and swift re-epithelization thus prevents infection and scarring.

#### Epithelial-to-Mesenchymal/Mesenchymal-to-Epithelial Transition

Epithelial-to-mesenchymal transition (EMT) is a key process underlying the normal course of re-epithelialization, ECM deposition and early presence of myofibroblast(-like) cells in wounds [95,96]. The process of EMT in keratinocytes is initiated by the loss of tight junctions followed by E-cadherin (epithelial marker) switch to N-cadherin (mesenchymal marker) [97]. Subsequently, epithelial cells dramatically remodel the cytoskeleton, including de novo expression of α-smooth muscle actin (α-SMA), vimentin and fibroblast-specific protein 1 [97,98], resulting in the escape of newly formed mesenchymal cells away from the epidermis to facilitate scarring/fibrosis. Crucial E-cadherin-repressing and EMT-controlling factors involve transcription factors such as snail and slug (zinc finger proteins Snai1 and Snai2—formerly known as Slug) [99] and twist (basic helix-loop-helix protein) [100]. Further, EMT profibrotic repressors of E-cadherin also include zinc finger E-box-binding homeobox (ZEB) 1 and ZEB2 [101]. At the beginning, the EMT-derived myofibroblasts [102] promote wound contraction and deposit ECM, and thus promote skin repair. However, later, in particular during the unresolved prolonged inflammation [11], the EMT is provoked to produce new generations of cells producing excessive ECM contributing to hypertrophic scarring. Morphologically, the epidermis located over a hypertrophic scar is thicker, the mesechymal markers are upregulated, and the basement membrane is depredated [103]. The persistent presence of α-SMA-expressing cells is thus typical of hypertrophic scars.

The in vitro 3D-organotypic full-thickness skin wound model [104] and a mouse model [105] revealed that only cells of the basal layer migrate in contact with the wound surface, whereas cells of the suprabasal layer move as a sheet without any dedifferentiation. The mouse study also identified the leading edge formed only of migrating keratinocytes followed by a proliferative hub containing both interfollicular and hair follicle-derived stem cells [105]. Asymmetric division of stem cells produces a high number of progenitor cells facilitating epidermis regeneration [106]. Re-epithelization is orchestrated by different signals produced by other cell populations, e.g., nitric oxide synthesized by macrophages, or a panel of growth factors such as EGF, HGF, bFGF, TGF-α, and TGF-β produced by fibroblasts [107,108]. The cell communication is bidirectional, and activated keratinocytes thus also influence surrounding fibroblasts, endothelial cells, melanocytes, and lymphocytes [109]. Migration and proliferation of keratinocytes is stopped by contact inhibition, when a stratified epidermis covers the entire skin defect [110]. Dermo-epidermal junctions are built up from the margins to the center and finally, basal keratinocytes turn back to the stationary phenotype with apical polarity [111].

Although data concerning wounded skin are limited, regulation of the stemness/differentiation of cells by EMT is complemented and counterbalanced by a reverse process, mesenchymal-to-epithelial transition (MET) [112]. EMT, as described above, is a well-studied process involved in fibrosis, embryonic development, as well as in various hallmarks of cancer involving invasion, metastasis, stemness, and chemo-resistance [113]. Whereas inhibition of EMT has been extensively studied to overcome drug resistance and tumor dissemination [114,115], targeting MET presents a novel approach in cancer therapy [116]. So far, several MET-inducing or epithelial phenotype-enhancing transcription factors have been identified in cancer cells. Examples include Grainyhead-like protein 2 homolog (GRHL2) [117], Ovo-like (OVOL) family members [118,119], GATA-binding factor 3 (GATA3) [120], Forkhead Box A1 (FOXA1), FOXA3 [121] and Kruppel-like factor 4 (KLF4) [122]. However, MET induction in fibroblasts has been poorly studied. Mesenchymal phenotype in fibroblasts is preserved by DNA methylation [123], activity of specific barrier kinases [124] and microRNAs [125]. Thus, it has been suggested that activation of the intrinsic MET program may enhance [126] efficiency of the fibroblast to epithelial cell reprogramming. Only a few transcription factors activating MET in dermal fibroblasts have been identified, namely OVOL2, a potent MET enhancer, which was shown to induce MET in cooperation with already known reprogramming factors HNF1 homeobox A (HNF1A), tumor protein p63 (TP63) and KLF4 [126].

### 3.2. Fibroblast, the Key Cell

It has been shown that dermal fibroblasts are a heterogeneous population of mesenchymal cells (Table 1). In practical terms, fibroblasts are often defined by a combination of their morphology, tissue position and absence of endothelial, epithelial and leukocyte markers, whereas the intermediate filament vimentin and platelet-derived growth factor receptor-α (PDGFRα) are typically present [127]. In parallel, CAFs have been defined by a specific subset of markers, including α-SMA, tenascin C, fibroblast-specific protein-1, fibroblast activating protein, and neural-glial antigen [128]. Morphologically, we have recognized at least two distinct subgroups of normal skin fibroblasts, i.e., papillary and reticular (Figure 2), originating from one common precursor [129]. However, detailed single cell sequencing revealed up to six subgroups of dermal fibroblasts. Out of the identified populations, only fibroblasts expressing dipeptidylpeptidase IV are able to produce ECM, which is fundamental for wound healing [130]. Accordingly, the extent of scar formation depends on the transcriptomic profile of fibroblasts present in the wounds [131].

**Figure 5:**
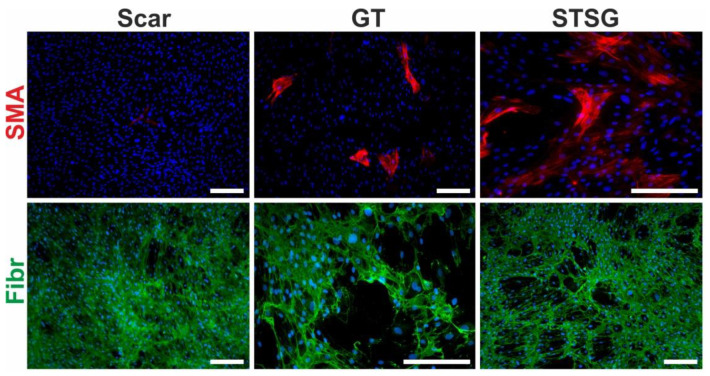
Primary cultures of human dermal fibroblasts isolated from matured scar (Scar), granulation tissue (GT) and split-thickness skin graft (STSG). Cultures were stained for α-smooth muscle actin (SMA, red signal), fibronectin (Fibr, green signal) and nucleus (DAPI, blue signal) (scale bar 100 µm).

During the first phase of skin repair, local fibrocytes/fibroblasts are activated (by inflammatory factors) and (chemo)attracted (by a gradient) into the injury site [132]. Many of them differentiate into α-SMA-expressing myofibroblasts (Figure 5) [133]. In addition, myofibroblasts may also be derived from other sources, for example, epithelial cells through the EMT, epidermal stem cells [134], bone marrow-derived mesenchymal stem cells [135], and from endothelial cells through the process called endothelial-mesenchymal transition [136].

#### ECM Deposition

Deposition and reorganization of ECM leading to scar formation are part of a normal healing process. Fibroblasts and myofibroblasts are stimulated by several cytokines, growth factors and/or growth adhesion regulatory galectins (e.g., TGF-β1, CTGF, PDGF, IGF, Gal-1, etc.). In detail, TGF-β1 acts as the critical cytokine in fibroblast-to-myofibroblast differentiation, while Gal-1 is rather responsible for myofibroblast activation. In this context, it has been shown that the expression of Gal-1, primarily localized to α-SMA-expressing myofibroblasts, increases substantially during skin wound healing and remains persistently elevated in hypertrophic scars [137]. They produce high amounts of structural components of the ECM (e.g., fibronectin, tenascin, collagen types III and I, etc.), which under normal circumstances are well regulated and their production decreases with the gradual overlap of proliferation phase to the maturation/remodeling phase of wound repair [138,139,140,141]. However, if the information is misregulated, for instance by prolonged inflammation in reticular dermis, abundant fibrotic tissue develops and a pathological scar is formed [142]. Collagen synthesis is sevenfold higher in hypertrophic scars (20-fold in keloids) compared to the normal skin wounds [13]. The collagen I to III ratio in hypertrophic scars is 6:1, whereas in normal skin it is 5:1 (17:1 in keloids). Furthermore, collagen cross-links most frequently occur in keloids (twice as much as in hypertrophic scars), resulting in formation of typical thick hyalinized eosinophilic collagen bundles not seen in hypertrophic scars. Hypertrophic scars exhibit nodular collagen of rather regular fiber thickness with its long axis parallel to the epidermis, similarly as may be seen in keloids. Of note, the α-SMA-expressing myofibroblasts are also variably expressed in both forms of scars [143]. Many other ECM components (e.g., fibronectin, hyaluronic acid, tenascin) are also increased in hypertrophic scars and keloids with certain variability [144].

### 3.3. Angiogenesis

Angiogenesis, development of new capillaries from preexisting vessels leading to microvascular network creation, is a crucial process in wound healing providing nutrients and oxygen to the damaged tissue [145]. Several angiogenesis stimulators have been discovered so far, namely, FGFα (FGF-1), bFGF (FGF-2), TGF-α, TGF-β, PGE2, TNF-α, VEGF, and EGF [146,147,148,149,150,151,152,153]. On the other hand, thrombospondin-1, connective tissue growth factor, tissue inhibitors of metalloproteinases, interferon-α, interferon-β, interferon-γ, angiostatin, and endostatin were identified as inhibitors of angiogenesis [145,154,155,156,157,158]. In burn wounds, however, the most potent stimulator of neovascularization is HIF-1 [159], as well as VEGF and CXCL12 (C-X-C motif chemokine 12 also known as stromal cell-derived factor 1) [160]. Moreover, angiogenesis in burned patients is sustained by an elevated blood level of endothelial progenitor cells corresponding to the size of traumatized area [161]. The best studied angiogenesis stimulator, VEGF, acts as a vascular endothelial cell mitogen, potent vasodilator and factor increasing microvascular permeability, as well as a regulator of endothelial integrins during vessel sprouting [147,162]. Sprouting requires the activity of MMPs regulating degradation of ECM surrounding the original vessel, so that the exposed tip cells may migrate into the newly formed granulation tissue forming a new sprout. On the downside, to compensate for the loss of the degraded ECM, the residing fibroblasts produce collagen leading to increased deposition of the scar tissue. VEGF was also shown to upregulate expression of adhesion molecules on the surface of endothelial cells, in addition to VEGF-mediated vasopermeabilization. This supports leukocyte extravasation [163,164], a process of immune cell passage through the vessel wall. From this point of view, VEGF also indirectly supports scar formation through recruitment of macrophages and mast cells, immune cells which were found to promote excessive scar formation vastly present in hypertrophic scars and keloids [76,164,165] by a mechanism remarkably similar to that of proinflammatory cytokines [166,167,168]. In addition, VEGF may act as a chemokine and directly activate macrophages due to VEGFR (receptors for vascular endothelial growth factor) expression on their surface [168]. Although evidence points to the VEGF involvement in the scar tissue formation [169,170,171,172,173,174]), the exact mechanism of how VEGF promotes/regulates the scar formation has not been completely understand. Data published so far suggest that anti-VEGF treatment could minimize scarring, yet this hypothesis needs to be further evaluated in animal models of hypertrophic scarring and human clinical trials.

## 4. Maturation and Remodeling Phase of Wound Healing

**Figure 6:**
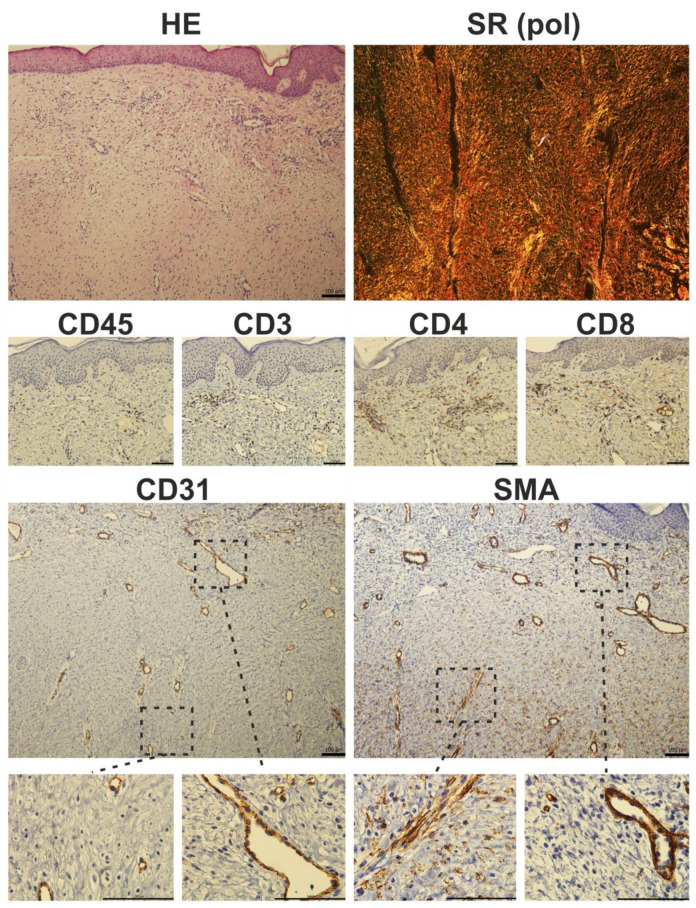
Histological photomicrograph of a hypertrophic scar stained with hematoxylin-eosin (HE) and Sirius red (SR) observed under polarized light (pol). Immunohistochemical staining of a hypertrophic scar. Prolonged inflammation results in the presence of CD45-positive cells in fibrotic tissue. CD3 staining showed characteristic T-cell islands, which were more pronounced after selective staining of CD4 and CD8-expressing T-cell subpopulations. The presence of myofibroblasts is restricted to deeper parts of the scar: in detail, α-smooth muscle actin (SMA) positivity was seen in luminized vessels (also positive for endothelial marker CD31), whereas CD31-negative and SMA-expressing cells (myofibroblasts) were located rather in the deeper parts of the scars (scale 100 µm).

It is well known that scar remodeling may last up to several years. Failure of the maturation and modeling phase may result in excessive collagen accumulation and formation of hypertrophic scars with enormous rigidity (Figure 6), or even in formation of a specific, not easily treatable unit—keloid. The connective tissue of keloid is morphologically similar to desmoplastic stroma of some tumors, especially of pancreatic adenocarcinoma [175]. In this context, fibroblasts isolated from keloids are very similar to cancer-associated fibroblasts isolated from the tumor stroma not only by expression of α-SMA, but also by expression of transcripts encoding inflammation-supporting cytokines and/or chemokines [176]. Due to the morphological similarity of keloids and tumors, cutaneous leiomyomas may sometimes be misdiagnosed as keloids and vice versa [177]. Of note, inhibitor of receptor kinase nintedanib, which is used in cancer therapy, is also effective in the treatment of therapy-refractive keloids [178].

If cells capable of generating mechanical forces are still present in activated state, contraction may graduate and results in poor cosmetic and/or functional deterioration of organs/tissues including the skin [179]. Furthermore, abnormal mechanical stresses in the wound microenvironment also induce hypertrophic scarring via activation of mechanotransduction pathways that include, but are not restricted to, integrin, Wingless-type, protein kinase B, and focal adhesion kinase, resulting in excessive cell proliferation, ECM formation, and finally fibrosis [180]. Fibroblasts are the main affected cell population, where mechanical force upregulates several profibrotic genes encoding proteins such as TGF-β1/2, α-SMA (Figure 6) and collagen I through different mechanoreceptors (integrins, growth factor receptors, G protein–coupled receptors, and ion channels). Therefore, it was not surprising to see that neutralization of TGF-β1/2 or exogenous addition of TGF-β3 reduced ECM deposition and scarring in a rat model [181]. Differentiated/activated myofibroblasts adhere to the ECM via cell surface receptors that transmit contractile forces leading to scar contracture [182]. Of note, the integrin–focal adhesion kinase (FAK) pathway plays a central role in the regulation of skin mechanotransduction. Mechanical forces during wound repair and/or scar formation activate the FAK pathway leading to intracellular signaling by numerous downstream factors (PI3K and MAPK kinases) mediating the fibrotic responses [183]. Thus, decreased FAK signaling was seen in nonhealing wounds, while FAK overactivation results in hypertrophic scar formation.

## 5. Conclusions

Wound healing represents a well-organized cascade of events that primarily aims to restore the barrier/protective function of the skin to avoid further blood loss and infection. Fibrosis and regeneration may be considered as counterparts regarding functional and cosmetic outcomes of wound repair. Normal wound healing is always a compromise between fibrosis and regeneration, carried out in a spectrum of events from hypertrophic repair, typical of young individuals following deep burns, to full regeneration seen in fetal wound healing. Therefore, complete understanding of the molecular mechanisms underlying the balance of fibrosis vs regeneration is the key to further therapy towards scarless healing in a fully controlled manner. Similarly, tumor growth and spreading is in many aspects similar to wound repair, as previously defined by Harold Dvorak [184,185]. The tumor vs wound parallel is also well documented in an example of signaling molecules [107]. From this point of view, study of both processes may be useful to better understanding of the biological issues related to poor healing in order to employ them in more efficient clinical practice. In addition, remarkable similarities between the behavior of keloid and cancer concerning their independent locally aggressive growth and resistance to treatment have been reported and their similarity at the cell/molecular biology level was shown in various features of cancer [186,187,188]. This relationship between both processes can be clearly demonstrated at the chemokine signaling level [189], of which the TGF-β family is the most distinguished representative [190].

Hence, signal cascades involving cell–matrix and cell–cell interactions in WME are crucial for future controlled clinical approaches preventing formation of excessive scars and directing the repair process towards regeneration, fully restoring the original functions of the skin.

## Figures and Tables

**Table 1:** Currently known differences between the main subpopulations of skin fibroblasts (− absent; + minimal; ++ mild; +++ moderate, ++++ marked, ? unknown).

Parameter/Cell Type	Papillary Fibroblasts 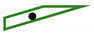	Reticular Fibroblasts 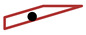	Hypertrophic Fibroblasts 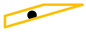	CAFs ^1^ 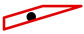
cell size	+	++	+	+
proliferation	++	+	++	+/−
collagen	++	++	++	++++
collagenase activity	++++	+	+	?
α-SMA	+	+++	+++	+++
collagen contraction	+	+++	+++	+++
TGF-β	+	+	+	+
TGF-β receptor 2	+	+++	+++	+/+++
CTGF	+	+++	+++	++++
osteopontin	+	+++	+++	+++
decorin	++++	+	+	+++
versican	+	+++	+++	++++

^1^ CAF—cancer-associated fibroblast.

## Abbreviations

aFGF	Acidic fibroblast growth factor
bFGF	Basic fibroblast growth factor
CAF	Cancer-associated fibroblast
CD	Cluster of differentiation
CTGF	Connective tissue growth factor
CXCL	Chemokine ligand
ECM	Extracellular matrix
EGF	Epidermal growth factor
EMT	Epithelial-mesenchymal transition
FAK	Focal adhesion kinase
Fibr	Fibronectin
FOXA	Forkhead Box A
Gal	Galectin
GRHL2	Grainyhead-like protein 2 homolog
GATA	guanine, adenine, thymine, adenine
GT	Granulation tissue
HGF	Hepatocyte growth factor
HIF	Hypoxia inducible factor
HNF1A	HNF1 homeobox A
IGF	Insulin growth factor
IL	Interleukin
IFN	Interferon
KLF4	Kruppel-like factor 4
MAPK	Mitogen-activated protein kinase
MET	Mesenchymal-to-epithelial transition
MMP	Matrix metalloproteinase
OVOL2	Ovo-like 2
PDGF	Platelet-derived growth factor
PGE2	Prostaglandin E2
PI3K	Phosphoinositide 3-kinase
ROS	Reactive oxygen species
SMA	Smooth muscle actin
STSG	Split thickness skin graft
TIMP	Tissue inhibitor of matrix metalloproteinase
TGF	Transforming growth factor
TNF	Tumor necrosis factor
TP63	Tumor protein p63
VEGF	Vascular endothelial growth factor
VEGFR	Vascular endothelial growth factor receptor
WME	Wound microenvironment
